# The MRZ reaction in primary progressive multiple sclerosis

**DOI:** 10.1186/s12987-016-0049-7

**Published:** 2017-02-07

**Authors:** Tilman Hottenrott, Rick Dersch, Benjamin Berger, Sebastian Rauer, Daniela Huzly, Oliver Stich

**Affiliations:** 1 Department of Neurology and Neurophysiology, University Medical Center Freiburg, Breisacher Strasse 64, 79106 Freiburg, Germany; 2 University Medical Center Freiburg, Institute of Virology, Hermann-Herder-Strasse 11, 79104 Freiburg, Germany; 3 Ravo Diagnostika GmbH, Oltmannsstrasse 5, 79100 Freiburg, Germany

**Keywords:** Primary progressive multiple sclerosis, PPMS, Intrathecal polyspecific antiviral immune response, MRZ reaction, MRZR, Oligoclonal bands, OCB

## Abstract

**Background:**

The MRZ reaction (MRZR), composed of the three antibody indices (AI) against measles, rubella and varicella zoster virus and found positive in the majority of relapsing-remitting multiple sclerosis (RRMS) patients, is absent in other inflammatory neurological diseases (OIND). So far, it has been uncertain whether its differential diagnostic promise extends to patients with primary-progressive multiple sclerosis (PPMS).

**Objective:**

To investigate the prevalence of MRZR in PPMS compared to RRMS and OIND patients.

**Methods:**

MRZR was assessed in patients with PPMS (n = 103), RRMS (n = 100) and OIND (n = 48). Both stringency levels for MRZR testing, MRZR-1 (≥1 AI positive) and MRZR-2 (≥2 AI positive), were applied.

**Results:**

Prevalence of positive MRZR-1 was 83.5% in PPMS and 67.8% in RRMS (p < 0.05). A positive MRZR-2 was found in 54.4% of PPMS and in 43.0% of RRMS patients (not significant). Compared to both MS subgroups, OIND patients exhibit lower frequencies of positive MRZR (MRZR-1: 22.9%, MRZR-2: 8.3%; p < 0.0001 each).

**Conclusion:**

Positive MRZR was at least as frequent in PPMS as in RRMS and much less frequent in OIND, confirming its promise as a potentially useful diagnostic tool for distinguishing both MS course types from OIND.

## Background

Multiple sclerosis (MS) is a chronic inflammatory autoimmune central nervous system (CNS) disorder displaying a variable disease course. At clinical onset, more than 85% of MS patients are classified as having relapsing-remitting MS (RRMS) [1]. These patients are predominately female and typically 20–30 years old at presentation of initial symptoms [1]. The remaining 10–15% of MS patients exhibit primary progressive MS (PPMS), characterised by a continuous worsening of symptoms from the onset, when patients are typically between 30 and 50 years old [1, 2]. PPMS affects men and women with similar frequency [1]. Both types of disease course exhibit inflammatory changes in the cerebrospinal fluid (CSF), e.g. the presence of oligoclonal bands (OCB), and inflammatory lesions in CNS detectable by magnetic resonance imaging (MRI) [1–3]. Diagnosis of PPMS and RRMS across the world today widely follows the 2010 revision of the McDonald criteria [4]. These require for PPMS a continuous disease progression of more than one year and that at least two of three additional criteria are met, one which is the presence of OCB in CSF. CSF analysis is no longer crucial for RRMS diagnosis, as here McDonald criteria primarily rely on clinical and MRI findings [4]. Despite their above mentioned similarities it is still a matter of debate whether PPMS and RRMS share sufficient underlying pathophysiological characteristics to be regarded as phenotypes of the same disorder and accordingly it cannot be taken as given that both will exhibit the MRZ reaction (MRZR) with similar frequency [3].

MRZR, first described in 1992, is a polyspecific, intrathecal humoral immune response to the three most frequent neurotropic viruses found in many RRMS patients: measles (M), rubella (R) and varicella zoster (Z), assessed using the three respective antibody indices (AI) [5]. A positive AI usually indicates intrathecal synthesis of antibodies against the respective pathogen, which is or recently was present in patient CSF. The absence of virus DNA in the CSF of MS patients with positive MRZ-AI led to the hypothesis of a ‘bystander reaction’, described as polyspecific B cell activation within the CNS [6]. While the pathophysiological role of MRZR may require further research, the interest in clinical practice is its potential to contribute to alternative diagnosis of MS. Whereas a positive MRZR is found in many RRMS patients, a few studies have shown negative MRZR in most patients with other autoimmune inflammatory neurological diseases (OIND) such as neuromyelitis optica (NMO), paraneoplastic neurological syndromes (PND), neurosarcoidosis (NS), acute disseminated encephalomyelitis (ADEM) and autoimmune encephalitis (AIE) [5, 7–9]. Additionally, MRZR has also been shown to be widely negative in patients with infectious CNS diseases which can mimic MS, such as Lyme neuroborreliosis and HTLV-I associated myelopathy [10, 11].

Studies showing a high prevalence of positive MRZR in MS to date are certainly fully applicable to RRMS; however the proportion of PPMS patients studied has been small, where any were included. Given current uncertainty about the similarity of PPMS and RRMS pathophysiology, and lack of understanding of the mechanisms linking MRZR to MS, our specific aim was to investigate MRZR in patients with PPMS.

Another limitation of previous studies has been the use of different criteria for positive MRZR. Some studies required only a single positive AI to constitute positive MRZR (MRZR-1), concluding that the prevalence of positive MRZR in MS is up to more than 90% [5], whereas others have required at least two AI positives for positive MRZR (MRZR-2), resulting in estimates of prevalence in MS of even below 50% [12].

This study investigated the prevalence of MRZR indicated by both the laxer MRZR-1 and the stricter MRZR-2 in a large cohort of PPMS patients. Results were compared to the prevalence of MRZR among patients with RRMS and some OIND.

## Methods

### Patients

This is a retrospective study in which all patients were treated at the University Medical Centre Freiburg in Germany between 2003 and 2015. Lumbar puncture (LP) had already been performed for all patients—for clinical purposes only and after written consent. CSF and serum samples were taken on the same day and stored according to consensus protocol for the standardization of CSF collection and biobanking [13]. Haemolytic CSF specimens were excluded. Diagnosis of MS was established according to the 2010 revised McDonald criteria, with particularly careful exclusion of relevant differential diagnoses [4]. All PPMS patients in the MS cohort were included for whom an MRZR measurement had been recorded or could be performed with existing CSF and serum samples. The MS cohort was screened for those with a recorded MRZR result, and from this set of MS patients, 100 RRMS patients were randomly drawn. The OIND group consisted of 48 patients for whom MRZR results were available from previous research, and unlike MS groups the OIND group did not include patients treated in 2015 [9]. Data concerning the ethnicity and immunization status of study patients was not available. The ethics committee of University Medical Centre Freiburg approved the study.

### MRZR and CSF analysis

All MRZR assessments were performed at the Department of Virology of the University of Freiburg between 2003 and 2015. All other CSF measurements relevant to this study were carried out in the CSF laboratory at the Department of Neurology. The CSF laboratory at the Department of Neurology regularly takes part in the External Quality Control of Diagnostic Assays and Tests (EQAS) for CSF diagnostics, including protein analytics, and the laboratory at the Department of Virology regularly takes part in the EQAS for virus diagnostics, including virus serology. Both EQAS procedures are performed by Instand e.V. (Gesellschaft zur Förderung der Qualitätssicherung in medizinischen Laboratorien e.V.; Düsseldorf; Germany).

Total immunoglobulin (Ig) concentrations in serum and CSF were detected nephelometrically (ProSpect System, Siemens, Germany), whereas measles-, rubella- and varicella-IgG (IgGspec) levels in CSF and serum were measured by enzyme linked immunosorbent assay (Serion *classic* ELISA, Germany), both according to the manufacturer’s instructions. MRZR was determined from the three respective virus-specific AI which were calculated as follows: AI = Q_IgG[spec]_/Q_IgG[total]_, if Q_IgG[total]_ < Q_lim_, and AI = Q_IgG[spec]_/Q_lim_, if Q_IgG[total]_ > Q_lim_ according to Reiber’s formula [14]. For a positive AI finding the threshold of AI ≥ 1.5 was applied [9, 10, 12, 15]. Previous studies have varied as to how many positive AIs are required for positive MRZR. In this study, MRZR-2 is used to refer to the MRZR definition requiring two or more positive AI, and MRZR-1 to refer to the MRZR definition requiring only one or more positive AI. Where an AI could not be calculated because no antibodies were detected in the CSF, AI was considered to be 1.0 (negative). CSF laboratory records were used which routinely include total CSF cell count, significant quantitative intrathecal antibody synthesis (defined as ≥10%), IgG index, QIgG, IgG concentration in CSF and presence or absence of oligoclonal bands (OCB) according to the Reibergrams and the CSF consensus report [16]. Detection of OCB for patients was performed using a highly sensitive isoelectric focusing technique on agarose gel followed by immunofixation (Hydragel Isofocusing, sebia, France) [17]. A positive OCB finding is defined as two or more OCB [16].

### Statistical analysis

Statistical testing of differences between groups on gender, prevalence of positive AI, MRZR, intrathecal Ig synthesis and OCB was performed using Fisher’s exact test (two-tailed). Differences of mean values of AI, total CSF cell count, intrathecal Ig synthesis, QIgG, IgG concentrations in CSF and age between groups were tested using Student’s *t* test (two-tailed). A p value <0.05 was regarded as statistically significant. The correlation between MRZR and OCB status was measured using the Phi correlation coefficient ($\phi$ ). A correlation coefficient between 0.2 and 0.4 was considered as weak, between 0.4 and 0.6 as intermediate and >0.6 as strong.

## Results

### Study population

The complete 2003–2015 cohort consisting of 1668 patients with a recorded MS diagnosis was retrospectively screened for MS subtype, RRMS or PPMS, following the 2010 McDonald criteria [4]. 236 PPMS patients (14.1%) were found of whom 96 had to be excluded due to missing CSF/serum samples and 37 due to insufficient clinical data, resulting in a PPMS group of 103 well-characterized patients. A random sample of 100 RRMS patients already tested for MRZR for clinical reasons was drawn from the same MS cohort. Additionally, an existing group of 48 patients with OIND was drawn on for comparison. Twenty-two of this OIND group had been diagnosed with neurosarcoidosis (NS), 19 with autoimmune encephalitis (AIE) and 7 with acute disseminated encephalomyelitis (ADEM). Table 1 shows key demographic features of the three study groups.

**Table 1:** Demographic data of all study patients

Study group	PPMS (n = 103)	RRMS (n = 100)	OIND (n = 48)	Comparison statistics
Gender, females in %	60.2	73.0	41.7	p < 0.05 for both MS groups vs. OIND; between MS groups: n.s.
Mean age in years at LP (range; SD)	51.3 (25–78; 10.0)	40.0 (19–74; 11.3)	51.8 (4–84; 18.4)	p < 0.0001 for PPMS and OIND vs. RRMS; between PPMS and OIND: n.s.

*PPMS* primary progressive multiple sclerosis, *RRMS* relapsing-remitting multiple sclerosis, *OIND* other autoimmune inflammatory neurological diseases comprising 22 patients with neurosarcoidosis (NS), 19 with autoimmune encephalitis (AIE) and 7 with acute disseminated encephalomyelitis (ADEM), *n* number of patients, *LP* lumbar puncture, *SD* standard deviation, *n.s.* not significant

### Virus-specific antibody indices (AI)

Results of AI tests of the three study groups are shown in Table 2. No statistically significant differences were found between the two MS groups in respect of frequency of one, two or three positive AIs and mean values of any of the three AI (M, R and Z). However, a positive AI for M and R was statistically significantly more frequent in PPMS compared to RRMS. Compared to both MS subgroups, the OIND group showed lower mean AI values and less frequent positive AI for all three viruses.

**Table 2:** Antibody indexes for all study patients

	PPMS (n = 103)	RRMS (n = 100)	OIND (n = 48)	Comparison statistics
Patients with 0 positive AI	16.5%	31.0%	77.1%	p < 0.0001 for both MS groups vs. OIND; p < 0.05 between MS groups
Patients with 1 positive AI	29.1%	26.0%	14.6%	all comparisons: n.s.
Patients with 2 positive AI	25.2%	25.0%	6.3%	p < 0.01 for both MS groups vs. OIND; between MS groups: n.s.
Patients with 3 positive AI	29.1%	18.0%	2.1%	p < 0.01 for both MS groups vs. OIND; between MS groups: n.s.
Positive AI for M	62.1%	48.0%	6.3%	p < 0.001 for both MS groups vs. OIND; p < 0.05 between MS groups
Positive AI for R	57.3%	43.0%	12.5%	p < 0.001 for both MS groups vs. OIND; p < 0.05 between MS groups
Positive AI for Z	48.5%	39.0%	14.6%	p < 0.05 for both MS groups vs. OIND; between MS groups: n.s.
Mean AI for M (range; SD)	3.3 (0.8–20.3; 3.2)	3.2 (0.6–52.2; 6.3)	1.0 (0.6–2.6; 0.3)	p < 0.01 for both MS groups vs. OIND; between MS groups: n.s.
Mean AI for R (range; SD)	3.1 (0.5–24.0; 4.0)	3.0 (0.5–19.8; 4.0)	1.2 (0.6-8.3; 1.2)	p < 0.01 for both MS groups vs. OIND; between MS groups: n.s.
Mean AI for Z (range; SD)	3.0 (0.6–19.8; 3.7)	2.5 (0.6–25.4; 3.5)	1.2 (0.4–3.8; 0.6)	p < 0.05 for both MS groups vs. OIND; between MS groups: n.s.

*PPMS* primary progressive multiple sclerosis, *RRMS* relapsing-remitting multiple sclerosis, *OIND* other autoimmune inflammatory neurological diseases, *positive AI* antibody index for measles (M), rubella (R) or varicella zoster (Z) ≥1.5, *n.s.* not significant

### MRZR

**Fig. 1:**
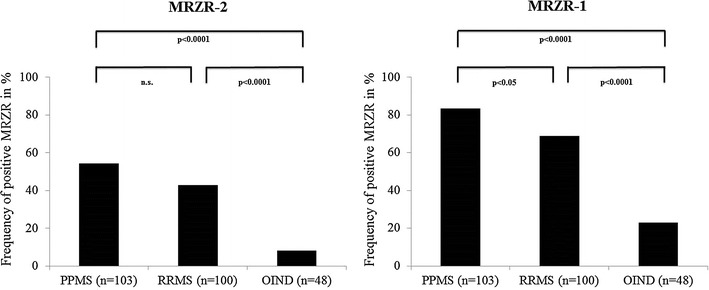
Frequency of positive MRZR-2 and MRZR-1 in patients with PPMS, RRMS and OIND. Frequency of positive MRZR-2 and MRZR-1 in patients with primary progressive multiple sclerosis (PPMS), relapsing-remitting multiple sclerosis (RRMS) and other autoimmune inflammatory neurological diseases (OIND). *MRZR-2* one or more positive AI, *MRZR-1* two or more positive AI, *n.s.* not significant

In accordance with AI findings, positive MRZR was found in a minority of OIND patients (MRZR-2: 8.3%, MRZR-1: 22.9%), statistically significantly less than in either of the MS subtypes (PPMS MRZR-2: 54.4%, PPMS MRZR-1: 83.5%; RRMS MRZR-2: 43.0%, RRMS MRZR-1: 69.0%—see Fig. 1).

Merging the two MS subtypes (PPMS and RRMS, n = 203) confirms that frequencies of both positive MRZR-2 (48.8%) and MRZR-1 (76.4%) among MS patients are statistically significantly different to those found in the OIND group (p < 0.0001 each).

### Comparison of MRZR results with previous studies

Table 3 presents MRZR results from the present study alongside those from previous studies. For these studies, findings from both MRZR definitions (MRZR-2 and MRZR-1) are listed where these could be reconstructed from the data provided in the relevant publication.

**Table 3:** A comparison of MRZR studies in multiple sclerosis patients

Study	Number of MS patients (MS subtypes)	Positive MRZR-2	Positive MRZR-1
*Present study*	n = 203 (PPMS: n = 103, RRMS: n = 100)	49%	76%
Reiber et al. [15]	n = 177 (no data concerning subtype)	[67%]	89%
Felgenhauer et al. [5]	n = 100 (no data concerning subtype)	[72%]	94%
Rosche et al. [24]	n = 68 (RRMS: n = 61, CIS: n = 7)	58%	[n.a.]
Brettschneider et al. [12]	n = 49 (CIS patients who converted to MS within 2 years)	47%	[n.a.]
Brecht et al.^a^ [19]	n = 46 (RRMS: n = 26, SPMS: n = 12, PPMS: n = 8)	[24%]	46%
Jarius et al. [7]	n = 42 (RRMS: n = 29, SPMS: n = 4, CIS: n = 9)	88%	[n.a.]
Bednarova et al. [10]	n = 42 (no data concerning subtype)	[47%]	88%
Hottenrott et al. [9]	n = 33 (RRMS: n = 14, SPMS: n = 5, PPMS: n = 14)	70%	[82%]
Kulakowska et al.^b^ [22]	n = 27 (RRMS: n = 21, PPMS: n = 6)	[56%]	81%
Tumani et al. [23]	n = 26 (no data concerning subtype)	[n.a.]	73%
Robinson-Agramonte et al. [21]	n = 23 (incomplete data concerning subtype)	[48%]	100%

The studies are presented in descending order of number of patients

Numbers in brackets were calculated by the author from data available in the respective article where possible

*MRZR-1* one or more positive antibody indices (AI) for measles (M), rubella (R) and varicella zoster (Z), *MRZR-2* two or more positive AI, *CIS* clinical isolated syndrome, *SPMS* secondary progressive multiple sclerosis, *PPMS* primary progressive multiple sclerosis, *RRMS* relapsing-remitting multiple sclerosis, *n.a.* not available

^a^This study investigated only OCB-negative MS patients

^b^This study included AI for HSV

### OCB

**Fig. 2:**
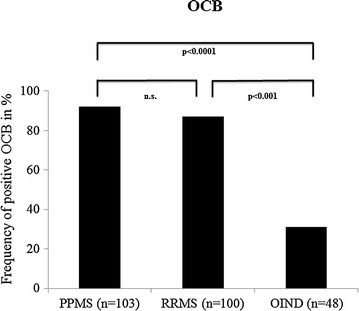
Prevalence of OCB in CSF of patients with PPMS, RRMS and OIND. Prevalence of oligoclonal bands (OCB) in cerebrospinal fluid (CSF) of patients with primary progressive multiple sclerosis (PPMS), relapsing-remitting multiple sclerosis (RRMS) and other autoimmune inflammatory neurological diseases (OIND), *n.s.* not significant

As can be seen from Fig. 2, the prevalence of positive OCB in CSF was similar in the two MS groups (93.2% for PPMS and 87.0% for RRMS) and significantly higher than in the OIND group (31.2%).

### OCB and MRZR

As presented in Tables 4 and 5, there was a weakly positive correlation between OCB and MRZR status (applying to both MRZR-2 and MRZR-1) in the combined MS group. Among the few OCB negative MS patients, 15.0 and 30.0% showed positive MRZR-2 and MRZR-1 respectively.

**Table 4:** Correlation between the presence of oligoclonal bands and MRZR-2 in patients with multiple sclerosis

Combined MS group (n = 203)	OCB positive	OCB negative		Association metrics
MRZR-2 positive	96	3	99	
MRZR-2 negative	87	17	104	
	183	20	203	*ϕ* = 0.22

*OCB positive* ≥2 oligoclonal bands in CSF, *MRZR-1* one or more positive antibody indices (AI) for measles (M), rubella (R) and varicella zoster (Z), *MRZR-2* two or more positive AI, *positive AI* AI ≥ 1.5, combined MS group: all primary progressive multiple sclerosis patients (n = 103) and relapsing-remitting multiple sclerosis patients (n = 100), *ϕ* Phi correlation coefficient

**Table 5:** Correlation between the presence of oligoclonal bands and MRZR-1 in patients with multiple sclerosis

Combined MS group (n = 203)	OCB positive	OCB negative		Association metrics
MRZR-1 positive	149	6	155	
MRZR-1 negative	34	14	48	
	183	20	203	$\phi$ = 0.36

*OCB positive* ≥2 oligoclonal bands in CSF, *MRZR-1* one or more positive antibody indices (AI) for measles (M), rubella (R) and varicella zoster (Z), *MRZR-2* two or more positive AI, *positive AI* AI ≥ 1.5, combined MS group: all primary progressive multiple sclerosis patients (n = 103) and relapsing-remitting multiple sclerosis patients (n = 100), *ϕ* Phi correlation coefficient

### CSF routine findings in MS patients

Inflammatory CSF changes were found in 93.2% of PPMS patients and in 87% of RRMS patients (n.s.). The most frequent pathological CSF finding was positive OCB (see above) followed by quantitative intrathecal IgG synthesis (51.5% in PPMS patients and 50.0% in RRMS; n.s.), elevated total CSF cell count (PPMS: 22.3%, RRMS: 43.0%; p < 0.01), quantitative intrathecal IgM synthesis (PPMS: 8.7%, RRMS: 14.8%; n.s.) and quantitative intrathecal IgA synthesis (PPMS: 7.8%, RRMS: 8.0%; n.s.). The data from the routine CSF analyses are presented in Table 6.

**Table 6:** Results of routine CSF analysis for MS patients

	PPMS (n = 103)	RRMS (n = 100)	Comparison statistics
Total CSF cell count in/µl (range; SD)	4.1 (1–43; 5.9)	8.9 (1–47; 10.3)	p < 0.001
Intrathecal IgG synthesis in % (range; SD)	19.8 (0–75.4; 21.8)	21.6 (0–84.3; 25.0)	n.s.
Intrathecal IgA synthesis in % (range; SD)	3.8 (0–54.9; 12.6)	2.1 (0–47.4; 7.5)	n.s.
Intrathecal IgM synthesis in % (range; SD)	5.0 (0–92.4; 17.0)	6.1 (0–88.3; 16.8)	n.s.
IgG index (range; SD)	0.93 (0.45–2.83; 0.42)	1.02 (0.23–4.0; 0.65)	n.s.
QIgG × 10^−3^ (range; SD)	5.3 (2.0–16.9; 3.1)	5.2 (1.0–15.8; 3.3)	n.s.
IgG concentration in CSF in mg/l (range; SD)	60.7 (19.4–217.0; 42.4)	55.9 (9.1–206.0; 39.8)	n.s.

*PPMS* primary progressive multiple sclerosis, *RRMS* relapsing-remitting multiple sclerosis, *IgG* immunoglobuline G, *IgA* immunoglobuline A, *IgM* immunoglobuline M, *QIgG* quotient of IgG, *SD* standard deviation, *n.s* not significant. All group values are expressed as means

## Discussion

To our knowledge, this is the first systematic investigation of the MRZR in a distinct PPMS cohort of reasonable size, and comparison with RRMS and OIND. The two main results are that positive MRZR is at least as frequent in PPMS as in RRMS patients and significantly more frequent than among OIND patients.

### Study population

PPMS patients showed typical demographic features: a more balanced gender distribution and a higher average age than RRMS patients [3, 18]. It has to be mentioned that diagnosis of RRMS can usually now be made earlier than at the mean age of this cohort (39 years). The high average age of RRMS patients might be a consequence of selection bias, as discussed below.

### AI and MRZR

Frequencies of positive AI and AI mean values in both MS groups (M > R > Z) were similar compared to a previous study but inverse in OIND patients (M < R < Z, Table 2) [15]. The rather low frequency of positive MRZR-2 in the OIND group indicates that MRZR-2 might be particularly helpful in distinguishing both MS subtypes from OIND. The less rigorous MRZR-1 is clearly less specific, showing more false positive results in OIND. This was also found in another recent study in tertiary care, where MRZ-1 was found to be positive in 19% of patients with OIND (well in line with the 23% in our OIND group) and 8% of patients with other non-inflammatory neurological diseases (OND), whereas none of these 53 patients were MRZR-2 positive [19]. Up to now, there is no indication of a pathophysiological role of any of the three MRZ viruses in MS pathogenesis, unlike in the case of the Epstein-Barr-virus (EBV) where it was shown that after EBV infection the risk of developing MS is increased [20].

Table 3 illustrates how inconsistent results of MRZR studies can appear if differences in definition (MRZR-2 vs. MRZR-1) are not considered. After clearly distinguishing between MRZR-2 and MRZR-1, the MRZR results from this study align quite well with most previous research. The study shows positive MRZR-1 in 76% of MS patients, which is less frequent than in some earlier studies [5, 10, 15, 21]. But, the frequency of positive MRZR-1 is closely in line with two other recent studies [22, 23]. Furthermore, several studies established a similar frequency of MRZR-2 positives of around 50% [10, 12, 21, 22, 24]. Exceptionally, Jarius et al. reported a considerably higher proportion of positive MRZR-2 (88%) in their MS cohort, which was used in two MRZR studies [7, 8]. Reasons for this divergence remain elusive, but selection bias is a conceivable explanation. Unfortunately, study reports did not include a detailed description of patient selection. In the present study, there may have been selection bias in the RRMS group, due to including patients with MRZR already previously performed for clinical reasons. Though such a bias might have contributed to their unexpectedly high mean age, this was actually similar to the mean age of MS patients in an even larger retrospective CSF study (not addressing MRZR) [25]. There is a lower risk of selection bias in the PPMS group because all available PPMS patients were included in the analysis, where possible. This is supported by the fact that our PPMS group matches demographic characteristics of PPMS patients studied elsewhere very well [1, 3, 18]. Limitations of this study include the monocenter cohort, the retrospective design and the lack of data concerning ethnicity and vaccination status of enrolled patients. Infection rates and vaccination status in respect of the three MRZ viruses can influence MRZR results, as has been demonstrated for the rubella virus in Cuba [21]. Therefore, verification of the MRZR results for PPMS patients with known vaccination status, and expansion to a multicenter prospective study, including populations from different parts of the world, would be a valuable next step.

### OCB and other CSF routine parameters

As expected, a high and very similar prevalence of OCB of around 90% was found in PPMS and RRMS patients- confirming the high sensitivity of this CSF parameter for both MS subgroups. The prevalence of OCB in PPMS and RRMS patients found here matches the findings of a previous monocenter Canadian study investigating 451 MS patients with CSF analyses performed between 1993 and 2007 [25]. In that study positive OCB was reported in 91% of RRMS and in 85% of PPMS patients.

Though the high prevalence of OCB in MS patients can be useful, a diagnostic disadvantage of OCB is their high prevalence in related conditions of diagnostic importance, in particular OIND. For example, OCB can be found in 40% of patients with neurosarcoidosis and in 50% of patients with neurologic complications of systemic lupus erythematosus (neuro-SLE) [9, 26].

The correlation between MRZR and OCB positivity in MS patients was weak, showing that MRZR provides additional, independent diagnostic information as previously reported for clinical isolated syndrome (CIS) [12]. One important issue in this context is whether MRZR tests can potentially improve the accuracy of diagnosis based on the 2010 revised McDonald criteria [4]. Referring to Tables 4 and 5, it appears that a combination of MRZR and OCB criteria could extend diagnostic sensitivity for the small proportion of OCB negative MS patients with 15% MRZR-2 positive and 30% MRZR-1 positive results. This seemingly contradicts findings in a group of 177 MS patients in which no patient was found MRZR positive and OCB negative [15]. This led the authors to recommend testing for MRZR only in OCB positive patients. However, the very high OCB prevalence in that study group (98%) meant that there were only three or four OCB negative patients, so that it could easily be a result of chance that none of these patients were found MRZR positive. MRZR/OCB findings of the present study are in line with a recent study in which MRZR-2 positives made up even 24% of 46 OCB negative MS patients [19]. Apart from that, MRZ-specific OCB were found in nearly 50% of MS patients without routine OCB [17]. Taking these results together, testing for MRZR in OCB negative patients with suspected MS appears well justified.

Considering the other CSF routine parameters, only the total cell count differed between the two MS subgroups: RRMS patients more often showed an elevated total cell count and a higher mean total cell count compared to PPMS patients. All Ig parameters (total IgG concentration in CSF, QIgG and intrathecal IgG/M/A synthesis) were similar between PPMS and RRMS patients paralleling the MRZR-2 results and indicating that these two MS subgroups seemingly display similar humoral immune responses in CSF.

## Conclusions

Positive MRZR has now been shown to be at least as frequent in PPMS patients as in RRMS, suggesting MRZR could be used in the diagnostic workup for both subtypes of MS in a similar way. MRZR has also been found to be less prevalent in OIND patients compared to OCB indicating that particularly the stricter MRZR-2 measure may well be helpful in the critical task of distinguishing OIND from MS. Altogether, these study results further support the significance of CSF analysis as an important tool to ensure alternative diagnoses of MS are promptly detected and properly treated [27].
